# Immunogenicity and efficacy of XBB.1.5 rS vaccine against EG.5.1 variant of SARS-CoV-2 in Syrian hamsters

**DOI:** 10.21203/rs.3.rs-3873514/v1

**Published:** 2024-02-09

**Authors:** Jacco Boon, Nadia Soudani, Traci Bricker, Tamarand Darling, Kuljeet Seehra, Nita Patel, Mimi Guebre-Xabier, Gale Smith, Mehul Suthar, Ali Ellebedy, Meredith Davis-Gardner

**Affiliations:** Washington University School of Medicine; Washington University School of Medicine; Washington University School of Medicine; Washington University School of Medicine; Washington University School of Medicine; Novavax, Inc.; Novavax, Inc; Novavax, Inc; Emory University School of Medicine; Washington University School of Medicine; Emory University School of Medicine

## Abstract

The continued emergence of SARS-CoV-2 variants necessitates updating COVID-19 vaccines to match circulating strains. The immunogenicity and efficacy of these vaccines must be tested in pre-clinical animal models. In Syrian hamsters, we measured the humoral and cellular immune response after immunization with the nanoparticle recombinant Spike (S) protein-based COVID-19 vaccine (Novavax, Inc.). We also compared the efficacy of the updated monovalent XBB.1.5 variant vaccine to previous COVID-19 vaccines for the induction of XBB.1.5 and EG.5.1 neutralizing antibodies and protection against a challenge with the EG.5.1 variant of SARS-CoV-2. Immunization induced high levels of spike-specific serum IgG and IgA antibodies, S-specific IgG and IgA antibody secreting cells, and antigen specific CD4 + T-cells. The XBB.1.5 and XBB.1.16 vaccines, but not the Prototype vaccine, induced high levels of neutralizing antibodies against XBB.1.5 and EG.5.1 variants of SARS-CoV-2. Upon challenge with the Omicron EG.5.1 variant, the XBB.1.5 and XBB.1.16 vaccines reduced the virus load in the lungs, nasal turbinates, trachea and nasal washes. The bivalent vaccine continued to offer protection in the trachea and lungs, but protection was reduced in the upper airways. In contrast, the monovalent Prototype vaccine no longer offered good protection, and breakthrough infections were observed in all animals and tissues. Thus, the protein-based XBB.1.5 vaccine is immunogenic and can protect against the Omicron EG.5.1 variant in the Syrian hamster model.

## INTRODUCTION

Severe acute respiratory syndrome coronavirus 2 (SARS-CoV-2) has caused hundreds of millions of infections worldwide and over 7 million deaths. Vaccines targeting the SARS-CoV-2 spike protein were developed within one year of the start of the pandemic, and they were remarkably effective in protecting against severe coronavirus disease 2019 (COVID-19), with efficacy rates ranging from 75 to 95% depending on the vaccine, the circulating strain, and age of the individual ^1–3^. In November of 2021, the Omicron variant of SARS-CoV-2 emerged, quickly spreading globally and replacing previous variants of concern (VOC) of SARS-CoV-2. Omicron variants harbor more than 30 amino acid substitutions in the spike (S) protein, which results in evasion of humoral immune responses and escape from protection of the original vaccines ^4^. The Omicron lineage of SARS-CoV-2 has continued to evolve away from neutralizing antibodies generated by previous infection or vaccination with ancestral vaccines, a process referred to as antigenic drift. Because of this drift, in 2022, global regulatory agencies recommended updating the COVID-19 vaccine to include the BA.5 variant of SARS-CoV-2. In late 2022, XBB-lineage Omicron variants of SARS-CoV-2 emerged and became successful ^5^. The XBB variants were resistant to antibodies induced by the BA.5 vaccine, prompting another update of the COVID-19 vaccine; the monovalent XBB.1.5 vaccine ^6^. Due to the continued evolution and drift of the SARS-CoV-2 virus, the ability of the updated vaccines to generate cross-protective immunity against future viral variants is crucial, and must be evaluated in preclinical animal models.

Novavax Inc. developed a SARS-CoV-2 recombinant S protein nanoparticle vaccine comprised of full-length prefusion S trimers co-formulated with a saponin-based adjuvant, Matrix-M^™^ (Prototype rS). In pre-clinical studies in mice and non-human primates, this vaccine was effective against a homologous challenge with SARS-CoV-2 ^7,8^. Similarly, in mice, a Beta (B.1.351 rS) version of this vaccine was effective against heterologous challenge with the Omicron BA.1 variant of SARS-CoV-2 ^9^. In Syrian hamsters, we showed that a boost with the BA.5 rS vaccine offered robust protection against a BA.5 virus challenge ^10^. In humans, immunization with the monovalent Prototype vaccine was effective against mild, moderate, or severe COVID-19 in clinical trials ^11–13^. Several trials reported the vaccine efficacy against symptomatic infection of 96% for the ancestral strain of SARS-CoV-2 and 86% for the alpha (B.1.1.7) variant ^13,14^. Boosting with a third or fourth dose of NVX-CoV2373 reduced the antigenic distance between the ancestral and Omicron BA.4/5 variants of SARS-CoV-2 ^15,16^, suggesting that repeated exposure to a subunit vaccine containing ancestral S protein induces a cross-reactive and cross-neutralizing antibody response.

Here, we evaluated the immunogenicity and efficacy of protein-based nanoparticle vaccines containing recombinant S proteins from Wuhan-1, BA.5, XBB.1.5, and XBB.1.16 variants of SARS-CoV-2 in hamsters. These vaccines induced robust S-specific cellular immune responses, S-specific IgG and IgA serum antibodies, and virus neutralizing antibodies in this pre-clinical animal model. Furthermore, the monovalent XBB.1.5 and XBB.1.16 vaccines provided protection against a heterologous challenge with the EG.5.1 variant of SARS-CoV-2.

## RESULTS

### Nanoparticle protein-based COVID-19 vaccine induces S protein specific IgG and IgA antibody secreting cells in Syrian hamsters.

**Figure 1:**
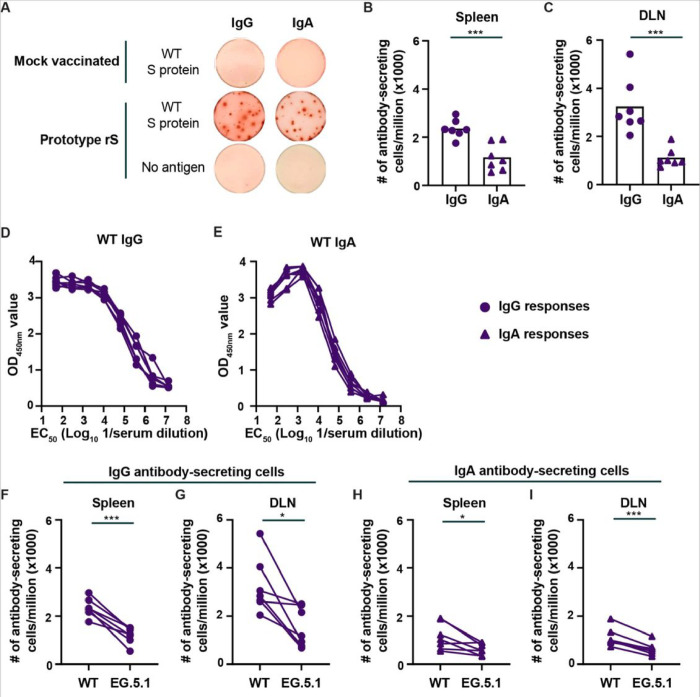
Prototype rS COVID-19 vaccine induces IgG and IgA secreting cells in the spleen and draining lymph node of hamsters. (**A**) Examples of IgG and IgA B-cell ELISpot data from control of Prototype rS immunized Syrian hamsters. S-specific IgG and IgA antibody secreting cells were detected in the draining inguinal lymph node of hamsters immunized three times with Prototype rS vaccine. (**B-C**) Frequency of Wuhan-1 (WT) S-specific IgG and IgA ASC /million in the spleen (**B**) and DLN (**C**) of hamsters 7 days after receiving a third dose of Prototype rS vaccine. (*** *P* < 0.001 by Student t-test). (**D-E**) Detection of WT S-specific IgG and IgA antibodies in hamster sera collected 21 days after receiving the second dose of Prototype rS vaccine. Presented are the OD_450nm_ values at different serum dilution. (**F-G**) Pairwise comparison of the frequency of WT and EG.5.1 S-specific IgG ASC/million in the spleen (**F**) and DLN (**G**) of hamsters 7 days after receiving a third dose of Prototype rS vaccine. (*** *P* < 0.001 and * *P* < 0.05 by pairwise comparison). (**H-I**) Pairwise comparison of the frequency of WT and EG.5.1 S-specific IgA ASC/million in the spleen (**H**) and DLN (**I**) of hamsters 7 days after receiving a third dose of Prototype rS vaccine. (*** *P* < 0.001 and * *P* < 0.05 by pairwise comparison). The results are from one experiment, and each symbol represents an individual animal (n = 7).

Groups of 5–6 week-old male Syrian hamsters (n = 7) were immunized intramuscularly twice at four-week intervals with 1 μg of the nanoparticle protein-based vaccine containing Prototype rS (Wuhan-1) and serum was collected 21 days later. Sixteen weeks later, the animals received a third dose of Prototype rS, and S-specific cellular responses were quantified 7 days later by B-cell and T-cell ELISpot. Compared to unvaccinated control animals, ~ 2,300 and ~ 3,200 Wuhan-1 (WT) S-specific IgG antibody secreting cells (ASC)/million were detected in the spleen and draining inguinal lymph nodes (DLN), respectively (Fig. 1A–C). In the same samples, we also detected WT S-specific IgA ASC, albeit the frequency per million cells was significantly reduced in the spleen (~ 1,150 ASC/million; *P* < 0.001) and DLN (~ 1,110 ASC/million; *P* < 0.001) compared to the frequency of IgG ASC (Fig. 1A–C). The ratio of IgG to IgA S-specific ASC was ~ 2.5:1 in both tissues 7 days after immunization. To support the presence of S-specific IgG and IgA ASC, an ELISA was performed on sera collected 21 days after the second immunization. Relatively high levels of S-specific IgG and IgA antibodies were detected in the serum of these animals (Fig. 1D–E).

To evaluate the impact of antigenic variation in the S protein on the B-cell cellular response, we compared the frequency of IgG and IgA ASC specific for WT and EG.5.1 S protein. A 1.8–2-fold reduction in the frequency of EG.5.1 S-specific IgG and IgA ASC was observed in the spleen (*P* < 0.001 and *P* < 0.05 for IgG and IgA respectively) and DLN (*P* < 0.05 and *P* < 0.001 for IgG and IgA respectively) of these animals (Fig. 1F–I).

### Immunization with a nanoparticle protein-based COVID-19 vaccine induces predominantly CD4 + T-cell response in Syrian hamsters.

**Figure 2:**
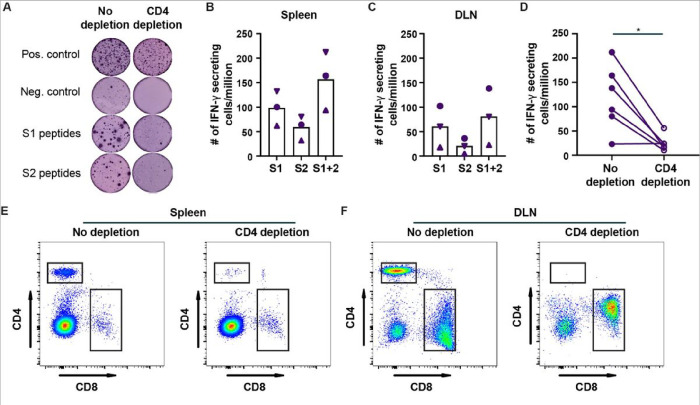
Prototype rS COVID-19 vaccine induces S-protein specific T-cell responses in Syrian hamsters. (**A**) Examples of an interferon-γ ELISpot using single cell suspensions obtained from the draining lymph node (DLN) of Syrian hamsters collected 7 days after immunization with a third dose of Prototype rS vaccine. Five hundred thousand cells were incubated with PMA + ionomycin (positive control), 1% DMSO (negative control), or pools of overlapping 15-mer peptides corresponding to the S1 and S2 subunit of the Spike protein of Wuhan-1 SARS-CoV-2. The same assay was performed after depletion of CD4+ cells. (**B-C**) Frequency of S1, S2 and S1+2 specific IFN-γ secreting cells/million detected in the spleen (**B**) and DLN (**C**) of Syrian hamsters 7 days after immunization with a third dose of Prototype rS vaccine. Different symbols correspond to different hamsters. (**D**) Comparison of the number of IFN-γ secreting cells in the spleen and DLN before and after CD4+ cell depletion. (* = *P* < 0.05 by paired t-test). (**E-F**) Flow cytometry plots conforming the depletion of CD4+ cells from the spleen (**E**) and DLN (**F**). The results are from one experiment with three animals.

We also determined the CD4 + and CD8 + T-cell response in the spleen and DLN of hamsters immunized three times with the Prototype rS vaccine by flow cytometry and interferon-gamma (IFN-γ) ELISpot assay. Flow cytometry analysis found that the average number of cells collected from the spleen and DLN was 50 and 36 million, respectively (**Fig S1**). Within the spleen, ~ 22% of the cells were CD4 + B220- cells and ~ 10% were CD8 + B220- cells. In the DLN, these frequencies doubled to ~ 40% and ~ 22% for the CD4 + and CD8 + T-cell population, respectively (**Fig S1**). Next, we measured the number of IFN-γ secreting cells/million following re-stimulation with pools of overlapping 15-mer peptides corresponding to the S1 and S2 subunit of Wuhan-1 SARS-CoV-2 Spike protein (Fig. 2A). Compared to our unstimulated negative control wells, re-stimulation with S1 or S2 subunit peptide pools induced IFN-γ secretion (purple spots in Fig. 2A). On average, we detected ~ 160 and ~ 80 IFN-γ secreting cells/million in the spleen and DLN of these hamsters, respectively (Fig. 2B–C). In all three hamsters, we detected more S1 specific cells compared to S2 specific IFN-γ secreting cells. To assess if the IFN-γ secretion was predominantly CD4 + or CD8 + cell mediated, we depleted the CD4 + cells *ex vivo*, confirmed depletion by flow cytometry (Fig. 2D–F) and quantified the number of S1 and S2 specific IFN-γ secreting cells in this CD4-depleted cell population. Depletion of CD4 + cells greatly reduced the number IFN-γ secreting cells detected by ELISpot assay (Fig. 2A and D). Overall, a 5-fold reduction (*P* < 0.05) in the number of IFN-γ secreting cells was detected in the spleen and DLN of these three hamsters (Fig. 2D). These data suggest that the nanoparticle protein-based vaccine induced a predominantly CD4-mediated T-cell response in hamsters.

### Nanoparticle protein-based XBB.1.5 and XBB.1.16 rS vaccines induce EG.5.1 specific neutralizing antibodies in Syrian hamsters.

**Figure 3:**
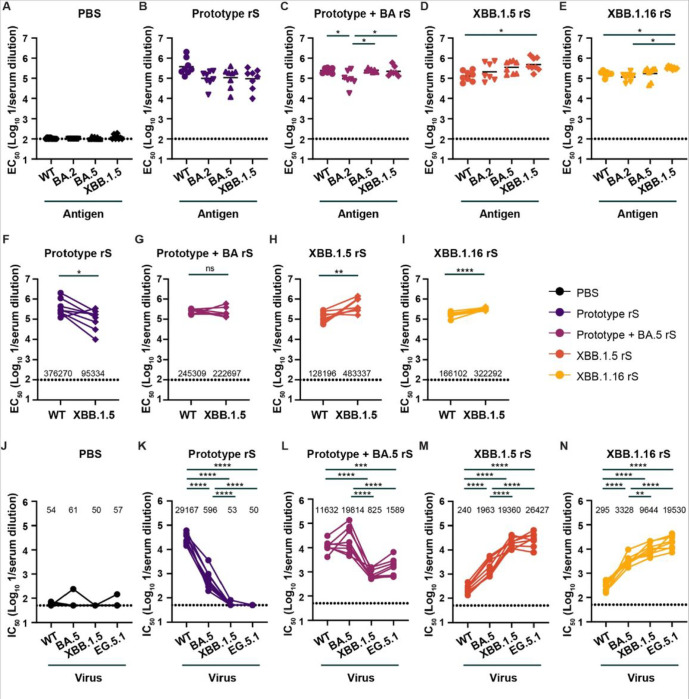
Humoral response following nanoparticle protein-based COVID-19 immunization in Syrian hamsters. (**A-E**) Serum anti-Wuhan-1 (WT), -BA.2, -BA.5, and -XBB.1.5 protein antibody response (EC_50_) in PBS control hamsters (**A**) or hamsters immunized twice with 1 μg of the Prototype rS (**B**), Prototype + BA.5 rS (**C**), XBB.1.5 rS (**D**), or XBB.1.16 rS (**E**) nanoparticle protein based vaccine. Serum was collected 21 days after the second dose of the vaccine. (* *P* < 0.05, by ordinary one-way ANOVA with a Tukey’s multiple comparisons corrections on ln-transformed EC_50_ values). (**F-I**) Pairwise comparison of the serum antibody titers (EC_50_) against Wuhan-1 (WT) or XBB.1.5 S protein measured by ELISA in the Prototype rS (**F**), Prototype + BA.5 rS (**G**), XBB.1.5 rS (**H**), or XBB.1.16 rS (I) immunized hamsters (**** *P* < 0.0001, *** *P* < 0.001, ** *P* < 0.01, ns = not significant by pairwise t-test). (**J-N**) Serum neutralizing antibody responses (IC_50_) against WA1/2020 (WT), BA.5, XBB.1.5, and EG.5.1 variant of SARS-CoV-2 in PBS control hamsters (**J**) or hamsters immunized twice with 1 μg of the Prototype rS (**K**), Prototype + BA.5 rS (L), XBB.1.5 rS (**M**), or XBB.1.16 rS (**N**) nanoparticle protein based vaccine. Serum was collected 21 days after the second and third dose of the vaccine. (**** *P* < 0.0001, *** *P* < 0.001, ** *P* < 0.01, by ordinary one-way ANOVA with a Tukey’s multiple comparisons corrections on ln-transformed IC_50_ values). Dotted line is the limit of detection. Animals at the limit of detection are arbitrarily assigned this value. These values are combined with those having values above the limit to determine the GMT. The results are from two experiments, and each symbol represents an individual animal (n = 8).

Groups of male hamsters were immunized twice at 4-week intervals with the nanoparticle protein-based vaccines containing the Prototype rS, Prototype + BA.5 rS, XBB.1.5 rS, or XBB.1.16 rS, or PBS as a control. Twenty-one days later, serum was collected and S-specific antibody responses were quantified by ELISA and virus neutralization assay. As expected, serum from control hamsters that received PBS did not bind to the S protein by ELISA (Fig. 3A). In comparison, serum collected from Prototype rS immunized hamsters contained high levels of anti-Wuhan-1 (WT) S-specific IgG (GMT of ~ 1:376,000) antibodies (Fig. 3B). Antibodies in sera from Prototype rS immunized hamsters also bound to S from BA.2 (~ 1:98,000), BA.5 (~ 1:108,000), and XBB.1.5 (~ 1:95,000) variant of SARS-CoV-2 (Fig. 3B). Sera obtained from hamsters immunized twice with the Prototype + BA.5 rS (bivalent) vaccine contained high IgG binding titers against S from WT (~ 1:245,000), BA.2 (~ 1:94,500), BA.5 (~ 1:249,000) and XBB.1.5 (~ 1:222,700). Significant difference in titer was observed between WT (*P* < 0.05), BA.5 (*P* < 0.05) and XBB.1.5 (*P* < 0.05) and the BA.2 titer (Fig. 3C). Immunization with XBB.1.5 rS induced high titers against XBB.1.5 (~ 1:483,000) and BA.5 (~ 1:352,000), with lower binding titers against BA.2 (~ 1:210,000) and WT (~ 1:128,000, *P* < 0.05) S (Fig. 3D). Similarly, immunization with XBB.1.16 rS induced high levels of IgG antibodies against XBB.1.5 (~ 1:322,000) and BA.5 (~ 1:173,000), but significantly lower titers against BA.2 (~ 1:115,000, *P* < 0.05) and WT (~ 1:166,000, *P* < 0.05) S (Fig. 3E). A pairwise comparison of serum IgG antibodies specific for WT (Wuhan-1) and XBB.1.5 S identified a 3.9-fold decrease (*P* < 0.05) in the Prototype rS immunized hamsters, no difference in the Prototype + BA.5 rS immunized hamsters and a 2–4 fold increase in the XBB.1.5 rS (*P* < 0.01) and XBB.1.16 rS (*P* < 0.001) immunized hamsters (Fig. 3F–I).

Serum samples were also tested for neutralization of SARS-CoV-2 by focus reduction neutralization test (FRNT) against WA/1/2020 (WT), BA.5, XBB.1.5 and EG.5.1 strains of SARS-CoV-2 (Fig. 3J–N). Whereas serum from the PBS control animals did not neutralize SARS-CoV-2, serum from immunized hamsters neutralized one or more strains of SARS-CoV-2 effectively (Fig. 3J–N). The serum neutralization titer against D614G was ~ 1:29,200 in the Prototype rS group, and this decreased significantly to 1:596 (*P* < 0.0001), 1:53 (*P* < 0.0001), and 1:50 (*P* < 0.0001) for the BA.5, XBB.1.5 and EG.5.1 strains of SARS-CoV-2 respectively (Fig. 3K). Two doses of the bivalent vaccine (Prototype + BA.5 rS) induced high serum neutralizing titers against the matched WT (~ 1:11,600) and BA.5 (~ 1:19,800) strains of SARS-CoV-2 (Fig. 3L). These sera also neutralized the XBB.1.5 (1:825) and EG.5.1 (1:1,589) variant of SARS-CoV-2, albeit the titer was significantly reduced compared to WT (*P* < 0.0001 for XBB.1.5; *P* < 0.001 for EG.5.1) and BA.5 (*P* < 0.0001 for XBB.1.5; *P* < 0.0001 for EG.5.1) virus. Immunization with XBB.1.5 rS induced high levels of neutralizing antibodies against the XBB.1.5 (1:19,360) and EG.5.1 (~ 1:26,400) variant of SARS-CoV-2, with significantly lower neutralizing titers against BA.5 (1:1,963, *P* < 0.0001), and WT (1:240, *P* < 0.0001) virus (Fig. 3M). Importantly, no significant difference in neutralization titers was detected between XBB.1.5 and EG.5.1 (*P* = 0.54) (Fig. 3M). Serum from XBB.1.6 rS immunized animals showed a similar pattern of neutralization with high titers against XBB.1.5 (~ 1:9,600) and EG.5.1 (1:19,530) variant of SARS-CoV-2 and significantly reduced titers against BA.5 (1:3,328, *P* < 0.0001) and WT (1:295, *P* < 0.0001) strain of SARS-CoV-2 compared to EG.5.1 neutralization titer (Fig. 3N).

### XBB.1.5 rS and XBB1.16 rS vaccines protect against an EG.5.1 virus challenge in Syrian hamsters.

**Figure 4:**
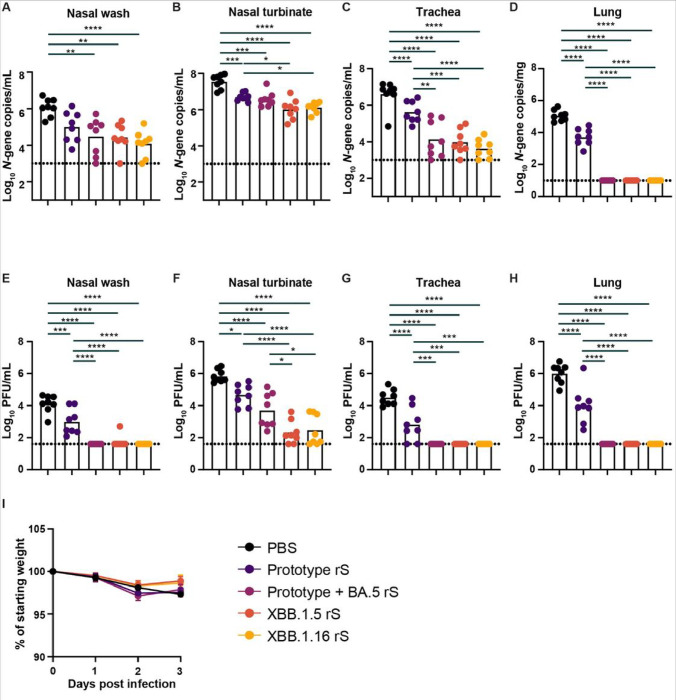
XBB.1.5 and XBB.1.16 rS vaccines protect against a heterologous challenge with the EG.5.1 variant of SARS-CoV-2 in Syrian hamsters. Prototype rS, Prototype + BA.5 rS, XBB.1.5 rS, XBB.1.16 rS immunized and control (PBS) Syrian hamsters were challenged with 10^4^ PFU of EG.5.1 variant of SARS-CoV-2 and nasal washes (**A and E**), nasal turbinates (**B and** F), tracheas (**C and G**), and lungs (**D and H**) were collected for analysis of viral RNA levels (**A-D**) and infectious virus (**E-H**) (**** *P* < 0.0001, *** *P* < 0.001, ** *P* < 0.01, * *P* < 0.05, ns = not significant by ordinary one-way ANOVA with a Tukey’s multiple comparisons corrections on ln-transformed data). (**I**) Morbidity or weight loss after EG.5.1 challenge. Bars indicate the geometric mean values, and dotted lines are the limit of detection of the assays. Animals at the limit of detection are arbitrarily assigned this value. The results are from two experiments, and each symbol represents an individual animal (n = 8).

Next, hamsters immunized twice with the Prototype rS, Prototype + BA.5 rS, XBB.1.5 rS, or XBB.1.16 rS, were challenged 11–12 weeks later intranasally with 1.0 × 10^4^ plaque forming-units (PFU) of EG.5.1 variant of SARS-CoV-2. This dose enables robust virus replication in the upper and lower respiratory tracts of hamsters and allow us measure differential efficacy of different vaccines and vaccine platforms ^17^. Animal weights were recorded daily for three days before nasal washes, nasal turbinates, trachea, and lungs were collected for virological analyses (Fig. 4). Compared to unvaccinated age and sex matched control animals, immunization with XBB.1.5 rS or XBB.1.16 rS significantly reduced viral RNA levels and infectious virus titers in the nasal wash, nasal turbinate, trachea and lungs of these animals (Fig. 4A–H). XBB.1.5 rS and XBB.1.16 rS reduced the amount of viral RNA in the nasal wash ~ 43-fold (*P* < 0.01) and 96-fold (*P* < 0.0001), and infectious virus titer ~ 240-fold (*P* < 0.0001) and ~ 325-fold (*P* < 0.0001) respectively (Fig. 4A and E). Similarly, in the nasal turbinates, immunization with XBB.1.5 rS or XBB.1.16 rS reduced the amount of viral RNA 33-fold (*P* < 0.0001) and 27-fold (*P* < 0.0001) and infectious virus titers 3,400-fold (*P* < 0.0001) and 2,500-fold (*P* < 0.0001) compared to the PBS control animals (Fig. 4B and F). The reduction in the amount of viral RNA was further increased to 422-fold (*P* < 0.0001) and 961-fold (*P* < 0.0001) in the trachea of XBB.1.5 rS or XBB.1.16 rS immunized hamsters (Fig. 4C). Also, no infectious virus was detected in the trachea of any of the hamsters (~ 800-fold and *P* < 0.0001 for both vaccines, Fig. 4G). No infectious virus was detected in the nasal wash or trachea of XBB.1.5 rS or XBB.1.16 rS immunized and EG.5.1 challenged hamsters (Fig. 4E and G). Finally, no viral RNA (~ 11,000-fold reduction, *P* < 0.0001) or infectious virus (25,000-fold, *P* < 0.0001) was detected in the lungs of XBB.1.5 rS or XBB.1.16 rS immunized and EG.5.1 challenged hamsters (Fig. 4D and H).

Syrian hamsters immunized twice with the bivalent Prototype + BA.5 rS vaccine also demonstrated significantly reduced amounts of viral RNA and infectious titers in the lungs, nasal wash and trachea with no discernible differences compared to the XBB.1.5 rS and XBB.1.16 rS immunized animals (Fig. 4). However, in the nasal turbinate, the amount infectious virus was significantly lower (*P* < 0.05) in the XBB.1.5 rS (~ 24-fold) and XBB.1.16 rS (~ 18-fold) immunized animals compared to hamsters that received the bivalent vaccine. Similarly, the amount of viral RNA was also lower (~ 3-fold), but this did not reach statistical significance. Finally, immunization with the Prototype rS vaccine significantly reduced the amount of viral RNA in nasal turbinates (~ 7-fold, *P* < 0.001), trachea (~ 9-fold, *P* < 0.0001), and lungs (~ 22-fold, *P* < 0.0001) compared to unvaccinated controls. It also reduced the amount of infectious virus in the nasal wash (~ 13-fold, *P* < 0.001), nasal turbinates (~ 15-fold, *P* < 0.05), trachea (~ 49-fold, *P* < 0.0001) and lungs (~ 97-fold, *P* < 0.0001) of these same animals. However, breakthrough infections were detected in all tissues tested in 100% of the animals. Compared to the Prototype + BA.5 rS, XBB.1.5 rS and XBB.1.16 rS immunized animals, protection from EG.5.1 challenge was greatly reduced in all four respiratory tissues of Prototype rS immunized animals (Fig. 4A–H). Combined, these data demonstrate the efficacy of the XBB.1.5 rS and XBB.1.16 rS vaccines and highlight the need for updating COVID-19 vaccines with contemporary variants of SARS-CoV-2 to more closely match newly emerging variants of SARS-CoV-2.

## DISCUSSION

In this study, we evaluated the immunogenicity of a nanoparticle protein-based COVID-19 vaccine in Syrian hamsters and compared the efficacy of the XBB.1.5 and XBB.1.16 variant vaccines to the original and bivalent COVID-19 vaccines, for protection against a challenge with the EG.5.1 variant of SARS-CoV-2. The nanoparticle protein-based subunit vaccine is highly immunogenic in Syrian hamsters and induced robust B- and T-cell responses against the Spike protein of SARS-CoV-2. Importantly, immunization with the XBB.1.5 or XBB.1.16 rS vaccine induced strong serum neutralizing antibody responses against XBB.1.5 and EG.5.1 variant of SARS-CoV-2. The antibody responses were associated with reduced viral burden after intranasal challenge with the EG.5.1 variant of SARS-CoV-2. Overall, these data demonstrate the efficacy of the XBB.1.5 vaccine against the novel EG.5.1 Omicron variant of SARS-CoV-2 in the pre-clinical hamster model of COVID-19.

The XBB.1.5 and XBB.1.16 rS vaccines induced robust XBB.1.5 and EG.5.1 specific antibodies capable of neutralizing both Omicron variants of SARS-CoV-2. Importantly, we did not observe a significant decrease in neutralization between XBB.1.5 and EG.5.1 despite the two amino-acid differences between the two strains (Phe456Lue and Gln52His). This observation is in line with previous studies in mice and non-human primates immunized twice with the XBB.1.5 rS or XBB.1.16 rS vaccine, or boosted once in pre-immune animals ^18^. In humans, the EG.5.1 variant was more resistant to neutralization compared to the XBB.1.16 virus in a cohort of individuals with a XBB breakthrough infection, albeit the different was less than 2-fold ^19^. However, a second study using convalescent sera from Prototype immunized and XBB variant infected individuals, did not detect any difference in neutralization between the XBB.1.5 and EG.5.1 virus ^20^. The high levels of neutralizing antibodies against EG.5.1 Omicron variant of SARS-CoV-2 were associated with a complete protection of the lower airways upon EG.5.1 challenge and a significant reduction in virus load in the upper airways. This is the first evidence in vivo that the XBB.1.5 vaccine can protect against EG.5.1 virus.

While the XBB.1.5 and XBB.1.16 rS vaccine induced significantly higher neutralizing antibody titers compared to the previous bivalent vaccines, immunization with the bivalent (Prototype + BA rS) vaccine did induce cross-neutralizing antibodies against XBB.1.5 and EG.5.1 Omicron variant SARS-CoV-2. The fold reduction in neutralization of the XBB.1.5 variant (~ 20-fold compared to BA.5 virus) was similar to that observed with a intranasal Chimpanzee adenovirus vectored bivalent vaccine in Syrian hamsters ^21^, suggesting that both vaccine platforms induce similarly broadly protective antibodies in this pre-clinical animal model. This highlights the need to periodically update the COVID-19 vaccine to better match contemporary and emerging variants of SARS-CoV-2. This data also demonstrates the power of the pre-clinical hamster model to be able to differentiate the vaccine efficacy between current and prior COVID-19 vaccines.

Despite the increasing utilization of hamsters in vaccine research, there is still a shortage of immunological tools specifically designed to evaluate immune responses in this model ^22^. Previous studies have highlighted the significance of T- and B-cells on SARS-CoV-2 infection and clearance in Syrian hamsters ^23–25^. In this study, we have developed the T- and B-cell ELISpot assay to provide the most complete immunogenicity analysis of a COVID-19 vaccine in Syrian hamsters to date. We show robust induction of S-specific IFN-γ secreting cells in the spleen and DLN of hamsters immunized with a protein-based vaccine, and discovered that the S-specific T-cell response was dominated by CD4 + cells in this setting. The frequency of IFN- γ secreting cells was on par with what was observed in mice and non-human primates that received the same vaccine ^18^. This study also showed for the first time, the induction of IgG and IgA ASC in the spleen and DLN following immunization with the Prototype rS vaccine. Importantly, we detected a ~ 2-fold reduction in the number of EG.5.1 variant specific ASC compared to the Wuhan-1 prototype S protein. This reduction in ASC coincided with a reduction in binding antibodies and a complete lack of neutralizing antibodies against the EG.5.1 variant of SARS-CoV-2. It also associated with a partial but significant loss of protection from a challenge with EG.5.1 variant of SARS-CoV-2.

### Limitations of the study.

We note several limitations of our study. (a) The vaccines were not tested in the context of pre-existing infection- or vaccine-induced immunity. While this would be valuable to investigate, we expect that all XBB.1.5 boosted animals will be fully protected against a challenge with the EG.5.1 variant of SARS-CoV-2 as was previously demonstrated by our group in a study that demonstrated that boosting mRNA vaccine-immunized hamsters with the bivalent Prototype + BA.5 rS vaccine conferred complete protection against the BA.5 variant of SARS-CoV-2 ^10^. (b) We did not evaluate the efficacy of each vaccine booster in male and female hamsters. Due to the number of variables (vaccines and time after vaccination), testing male and female animals in each experiment was not feasible. (c) B- and T-cell responses after immunization with variant vaccines like XBB.1.5 rS or XBB.1.16 rS were not measured. We expect that the T-cell response will be similar between Prototype rS and XBB.1.5 rS vaccine due to the limited variability of the S protein outside of the receptor binding domain. Similarly, and based on the serum antibody responses to XBB.1.5 and EG.5.1 in the XBB.1.5 rS immunized hamsters, we expect the XBB.1.5 rS vaccine to induce both IgG and IgA ASC and that they cross-react with the EG.5.1 variant. (d) Studies with more recent emerging variants (*e.g*., BA.2.86) are warranted. (e) The impact on virus transmission was not evaluated. While EG.5.1 can transmit between naïve hamsters, airborne transmission is not as effective as was observed for pre-Omicron variants of SARS-CoV-2 ^26^.

Overall, our studies demonstrate that nanoparticle protein-based vaccines are immunogenic and that the XBB.1.5 rS vaccine is effective against newer variants of SARS-CoV-2 in Syrian hamsters.

## STAR METHODS

### RESOURCE AVAILABILITY

#### Lead contact.

Further information and requests for resources and reagents should be directed to the Lead Contact, Adrianus C.M. Boon (jboon@wustl.edu).

#### Materials availability.

All requests for resources and reagents should be directed to the Lead Contact author. This includes viruses, vaccines, and primer-probe sets. All reagents will be made available on request after completion of a Materials Transfer Agreement.

#### Data and code availability.

All data supporting the findings of this study are available within the paper and are available from the corresponding author upon request. This paper does not include original code. Any additional information required to reanalyze the data reported in this paper is available from the lead contact upon request.

### EXPERIMENTAL MODEL AND SUBJECT DETAILS

#### Cells and Viruses.

Vero cells expressing human angiotensin converting enzyme 2 (ACE2) and transmembrane protease, serine 2 (TMPRSS2) (Vero-hACE2-hTMPRSS2 ^27,28^, gift from Adrian Creanga and Barney Graham, National Institute of Health) were cultured at 37°C in Dulbecco’s Modified Eagle medium (DMEM) supplemented with 10% fetal bovine serum (FBS), 10 mM HEPES (4-(2-hydroxyethyl)-1-piperazineethanesulfonic acid, pH 7.3), 100 U/mL Penicillin, 100 μg/mL Streptomycin, and 10 μg/mL of puromycin. Vero cells expressing TMPRSS2 (Vero-hTMPRSS2) ^28^ were cultured at 37°C in DMEM supplemented with 10% fetal bovine serum (FBS), 10 mM HEPES (pH 7.3), 100 U/mL Penicillin, 100 μg/mL Streptomycin, and 5 μg/mL of blasticidin.

The SARS-CoV-2 WT strain (2019-nCov/USA-WA1/2020) was obtained from BEI, the BA.5 variant of SARS-CoV-2 (hCOV-19/USA/COR-22–063113/2022) was a gift from R. Webby (St. Jude Children’s Research Hospital), the XBB.1.5 variant (hCoV-19/USA/MD-HP40900-PIDYSWHNUB/2022) was a gift from Andy Pekosz, and the EG.5.1 variant (hCoV-19/USA/CA-Standford-147_S01/2023, GISAID # EPI_ISL_17977757) was from M. Suthar. All viruses were propagated on Vero-hTMPRSS2 cells. The virus stocks were subjected to next-generation sequencing, and the S protein sequences were identical to the original isolates. The infectious virus titer was determined by plaque and focus-forming assay on Vero-hACE2-hTMPRSS2 or Vero-hTMPRSS2 cells.

#### Recombinant proteins.

Prototype recombinant S was expressed as previously described ^7,29^. SARS-CoV-2 rS, construct BV2373, is a recombinant nanoparticle vaccine constructed from the full-length, wild-type SARS-CoV-2 spike glycoprotein (GenBank accession number, MN908947; nucleotides 21563–25384). The native full-length S protein was modified by mutation of the putative furin cleavage site RRAR to QQAQ (3Q) located within the S1/S2 cleavage domain to be protease resistant. Two additional proline amino acid substitutions were inserted at positions K986P and V987P (2P) within the heptad repeat 1 (HR1) domain to stabilize SARS-CoV-2 S in a prefusion conformation, which is believed to optimize presentation of neutralizing epitopes. The BA.5 rS variant vaccine (construct BV2540) sequence was obtained from the GISAID database (EPI_ISL_12097410.1). To produce construct BV2540, the native full-length S protein was subjected to mutations applied to the ancestral Wuhan-Hu-1 rS plus additional mutations: V3G, T19I, A27S, G142D, V213G, G339D, S371F, S373P, S375F, T376A, D405N, R408S, K417N, N440K, L452R, S477N, T478K, E484A, F486V, Q498R, N501Y, Y505H, D614G, H655Y, N679K, P681H, N764K, D796Y, Q954H, N969K and deletions: ΔL24, ΔP25, ΔP26, ΔH69, ΔV70. The XBB.1.5 variant vaccine (construct BV2601) sequence was obtained from the GISAID database (EPI_ISL_16343574). To produce these constructs, in addition to the 3Q-2P mutations applied to the Prototype Wuhan-Hu-1 rS, the following mutations were introduced to the native full-length S protein: T19I, A27S, V83A, G142D, H146Q, Q183E, V213E, G252V, G339H, R346T, L368I, S371F, S373P, S375F, T376A, D405N, R408S, K417N, N440K, V445P, G446S, N460K, S477N, T478K, E484A, F486P, F490S, Q498R, N501Y, Y505H, D614G, H655Y, N679K, P681H, N764K, D796Y, Q954H, and N969K, as well as Δ24–26 and ΔY144. To produce the XBB.1.16 rS vaccine (construct BV2633, the GISAID database (EPI_ISL_17351426), in addition to the mutations applied to the Prototype Wuhan-Hu-1 rS to produce the BV2601 construct the following mutations were introduced to the native full-length S protein: K986P, V987P, E180V, and K478R. The synthetic transgenes were engineered into the baculovirus vector for expression in *Spodoptera frugiperda* (Sf9) insect cells. Prototype rS, Prototype + BA.5 rS, XBB.1.15 rS, and XBB.1.16 rS were formulated with Matrix-M adjuvant and stored at 2–8°C.

#### Hamster experiments.

Animal studies were carried out in accordance with the recommendations in the Guide for the Care and Use of Laboratory Animals of the National Institutes of Health. The protocols were approved by the Institutional Animal Care and Use Committee at the Washington University School of Medicine (assurance number A3381–01).

##### Immunogenicity analysis.

Seven five-week old male hamsters were obtained from Charles River Laboratories and housed at Washington University. Five days after arrival, the animals were immunized via intramuscular injection in the posterior thigh muscles with 1 μg of the protein nanoparticle Prototype rS in 100 μL (50 μL per leg), and 21 days later they were boosted with 1 μg of the same vaccine. Serum was collected 21 days later for the detection of S-specific IgG and IgA by ELISA. After 112 days, the animals received a third dose of the Prototype rS vaccine and 7 days later, the animals were euthanized, and the spleen and draining inguinal lymph nodes (DLN) were collected into 15 mL tubes containing 5 mL of ice-cold RPMI-1640 media with 2% FBS (R2). To generate a single cell suspension from the spleen or lymph nodes, the tissues were mashed using the plunger of a 1 mL syringe and filtered through a sterile 70 μm cell strainer. The cells were spin down at 300 × g for 5 min at 4 °C and red blood cells were lysed with 500 μL RBC lysis buffer (BioLegend) for 1 minute at room temperature. Next, 10 mL of R2 media was added, the cells were spin down, and resuspended in 1 mL ice-cold RPMI-1640 / 10% FBS (R10). Live and dead cells were counted using Acridine orange (AO) and propidium iodide (PI) (Sigma) using a cell counter (Nexcelom Bioscience), and the cells were diluted in R10 to a concentration of 10^7^ cells/mL and used for flow cytometry analysis, and T- and B-cell ELISpot analysis.

##### Vaccine efficacy analysis.

Five-week old male hamsters were obtained from Charles River Laboratories and housed at Washington University. Five days after arrival, the animals were immunized via intramuscular injection with 1 μg of the protein nanoparticle Prototype rS, Prototype + BA.5 rS (bivalent), XBB.1.5 rS, or XBB.1.16 rS vaccine. Control animals received PBS alone. Serum samples were obtained 21 days later and one week later the animals were immunized with a second dose of the same vaccine, and serum was collected 21 days later. Approximately two months later (day 59), the animals were randomly divided into two groups and one group was transferred to the enhanced Biosafety level 3 laboratory and challenged via intranasal route with 1 × 10^4^ PFU of Omicron EG.5.1 variant. The second group followed a week later and was also challenged with 1 × 10^4^ PFU of the EG.5.1 variant. Animal weights were measured daily for the duration of the experiment. Three days after challenge, the animals were necropsied, and their lungs, trachea, and nasal turbinates were collected for virological analysis. These tissues were homogenized in 1 mL of DMEM, clarified by centrifugation (1,000 × *g* for 5 min) and used for viral titer analysis by quantitative RT-PCR (RT-qPCR) using primers and probes targeting the *N* gene, and by plaque assay. A nasal wash was also collected, by inoculating 1 mL of PBS with 0.1% bovine serum albumin into one nostril and collecting the wash from the other nostril. The nasal wash was clarified by centrifugation (2,000 × *g* for 10 min) and used for viral titer analysis by RT-qPCR using primers and probes targeting the *N* gene, and by plaque assay.

### METHOD DETAILS

#### Focus reduction neutralization titer assay (FRNT).

Serial dilutions of serum samples, starting at 1:60, were incubated with 10^2^ focus-forming units (FFU) of different strains of SARS-CoV-2 for 1 h at 37°C. Antibody-virus complexes were added to Vero-hTMPRSS2 cell monolayers in 96-well plates and incubated at 37°C for 1 h. Subsequently, cells were overlaid with 1% (w/v) methylcellulose in Eagle’s Minimal Essential medium (MEM, Thermo Fisher Scientific). Plates were fixed 30 h (WA1/2020 and B.1.351) or 50 h (BA.5, XBB.1.5, and EG.5.1) later with 10% formalin in PBS for 20 min at room temperature. The increase in incubation time for the Omicron variants of SARS-CoV-2 is due to slower replication kinetics. Overlay and formalin were aspirated and plates were washed and sequentially incubated with a pool of anti-S murine antibodies (SARS2–02, −08, −09, −10, −11, −13, −14, −17, −20, −26, −27, −28, −31, −38, −41, −42, −44, −49, −57, −62, −64, −65, −67 and −71 ^30^ and HRP-conjugated goat anti-mouse IgG (Sigma Cat # A8924) in PBS supplemented with 0.1% saponin and 0.1% bovine serum albumin. SARS-CoV-2-infected cell foci were visualized using TrueBlue peroxidase substrate (KPL) and quantitated on an ImmunoSpot microanalyzer (Cellular Technologies).

#### Virus titration assays.

Plaque assays were performed on Vero-hACE2-hTRMPSS2 cells in 24-well plates. Homogenates of lungs, trachea and nasal turbinates, and nasal washes were diluted serially by 10-fold, starting at 1:10, in cell infection medium (DMEM + 2% FBS + 100 U/mL of penicillin-streptomycin). Two hundred and fifty microliters of the diluted virus were added to a single well per dilution per sample. After 1 h at 37°C, the inoculum was aspirated, the cells were washed with PBS, and a 1% methylcellulose overlay in MEM supplemented with 2% FBS was added. Ninety-six hours after virus inoculation, the cells were fixed with 10% formalin, and the monolayer was stained with crystal violet (0.5% w/v in 25% methanol in water) for 30 min at 20°C. The number of plaques were counted and used to calculate the plaque forming units/mL (PFU/mL).

To quantify viral load in lung tissue homogenates and nasal washes, RNA was extracted from 100 μL samples using the MagMax Viral Pathogen Kit (ThermoFisher) on the KingFisher Flex Purification System following the manufacturer’s protocol and eluted with 50 μL of water. Four microliters RNA was used for real-time RT-qPCR to detect and quantify *N* gene of SARS-CoV-2 using TaqMan^™^ RNA-to-CT 1-Step Kit (Thermo Fisher Scientific) as described ^31^ using the following primers and probes: Forward: GACCCCAAAATCAGCGAAAT; Reverse: TCTGGTTACTGCCAGTTGAATCTG; Probe: ACCCCGCATTACGTTTGGTGGACC; 5’Dye/3’Quencher: 6-FAM/ZEN/IBFQ. Viral RNA was expressed as *N* gene copy numbers per mg for lung tissue homogenates or mL for nasal washes, nasal turbinates, and trachea based on a standard included in the assay, which was created via *in vitro* transcription of a synthetic DNA molecule containing the target region of the *N* gene.

#### ELISA.

Ninety-six-well microtiter plates (Nunc MaxiSorp; ThermoFisher Scientific) were coated with 100 μL of recombinant SARS-CoV-2 S protein (Wuhan-1 strain, BA.2, BA.5, or XBB.1.5, generated by Novavax as described above) at a concentration of 1 μg/mL in PBS (Gibco) at 4°C overnight; negative control wells were coated with 1 μg/mL of BSA (Sigma). Plates were blocked for 1.5 h at room temperature with 280 μL of blocking solution (PBS supplemented with 0.05% Tween-20 (Sigma) and 10% FBS (Corning)). The sera were diluted serially in blocking solution, starting at 1:100 dilution and incubated for 1.5 h at room temperature. The plates were washed three times with T-PBS (1X PBS supplemented with 0.05% Tween-20), and 100 μL of HRP-conjugated anti-hamster IgG(H+L) antibody (Southern Biotech Cat. #6061–05) diluted 1:1000 in blocking solution, was added to all wells and incubated for 1 h at room temperature. Alternatively, plates were incubated with biotinylated anti-hamster IgA antibody (Brookwood Biomedical, Cat. # sab3002a) diluted 1:1000 in blocking solution for 1 hours, followed by three washes with T-PBS and 1:5000 diluted HRP-conjugated streptavidin (Zymed). Plates were washed 3 times with T-PBS and 3 times with 1X PBS, and 100 μL of 1-step Ultra TMB-ELISA substrate solution (Thermo Fisher Scientific) was added to all wells. The reaction was stopped after 10 min using 100 μL of 1N H_2_SO_4_, and the plates were analyzed at a wavelength of 450 nm using a microtiter plate reader (BioTek).

#### B-cell ELISpot assay.

Enzyme-linked immune absorbent spot (ELISpot) assays were performed to determine the number of S-specific IgG and IgA ASC ELISpot Multiscreen Filter Plates (Millipore) were coated overnight at 4°C with 1 μg/mL of rS from the Wuhan-Hu-1 or EG.5.1 strains of SARS-CoV-2. Control plates were either coated with anti-Syrian hamster IgG (1:100, Jackson ImmunoResearch) or left uncoated. The next day, the plates were blocked for 60 min at 37°C with RPMI 1640 supplemented with 10% FBS. Single cell suspensions of freshly isolated spleen or DLN cells (500,000 cells/well) were added in duplicate to the first row followed by 3-fold serial dilution of the cells. After 6 hours at 37°C, the cells were washed off, and secreted hamster IgG or IgA were detected with a biotinylated anti-Syrian hamster IgG (1:1000, Jackson ImmunoResearch) or anti-Syrian hamster IgA (1:1000, Brookwood Biomedical) detection antibody respectively. Following overnight incubation at 4°C, the plates were washed 3x with T-PBS and streptavidin-conjugated horseradish peroxidase (HRP, Invitrogen) diluted 1:5000 in PBS was added for 1.5 hours at RT. Following another three washes with T-PBS and 1 wash PBS, the plates were developed, and spots were formed through an enzymatic reaction in the presence of 3-Amino-9-Ethyl Carbazole (AEC) and H_2_O_2_ (Sigma). ELISpot plates were analyzed using an ELISpot counter (Cellular Technology Limited). Each spot represents an individual ASC and the number of spots indicates the frequency of B cells in the original sample that produces antibodies against the target antigen.

#### T-cell ELISpot assay.

Interferon-gamma (IFN-γ) ELISpot was done according to ELISpot Flex: Hamster IFN-γ kit (MABTECH) specifications. Briefly, the Polyvinylidene difluoride (PVDF)-lined microplates (Millipore) were coated overnight at 4°C with an IFN-γ capture antibody diluted in PBS (15 μg/ ml). Prior to the addition of cells, the wells were washed 5 times with PBS. A total of 500,000 cells in R10, were incubated peptide pools (10 μg/mL) of 15-mer overlapping peptides (BEI-Resources) corresponding to the S1 (1–668) and S2 (659–1273) subunit of S, PMA (phorbol myristate acetate, 0.5 μg/mL) plus ionomycin (1μg/mL) as a positive control, or 1% DMSO as a negative control. After 24 hours, the cells were washed off with PBS and the plates were incubated with 1 μg/mL of biotinylated IFN-γ-specific detection antibody in PBS-0.5% FBS for 2 hours at room temperature. Following another washing step 5 times with PBS the plates were incubated for 1 hours with streptavidin-conjugated alkaline phosphatase (ALP, 1:1000) in PBS-0.5% FBS. After washing 5x with PBS, BCIP/NBT substrate was added until the spots appeared. The color development was stopped by washing the plates extensively with water. ELISpot plates were analyzed using an ELISpot counter (Cellular Technology Limited).

#### CD4+ cell depletion.

CD4+ cell depletion was performed on cells collected from the spleen or draining lymph nodes using Dynabeads^™^ Biotin Binder kit (Invitrogen) containing magnetic beads. In short, the beads were washed twice with 2% FBS in PBS (P2). As per manufacturer, 50 μL of pre-washed beads were incubated with 10 μg/mL of biotinylated anti-CD4 (GK1.5, BioLegend) for 45 minutes at room temperature. The beads were washed 5 times with P2 and added to one million cells from the spleen or draining lymph node. The mixture was incubated for 30 min on ice with occasional shaking. Using the magnetic stand, the CD4+ cells were removed from the cell population and used for Flow cytometry and ELISpot assay.

#### Flow cytometry.

Staining was performed on the supernatant of CD4-depleted cells or 1x106 of non-depleted cells from the spleen or lymph node. The cells were stained for 30 min on ice with CD4-PE (GK1.5, 1:100, BioLegend), CD8b-BB700 (341, 1:100, BD Biosciences), B220-PE/Cyanine7 (RA3–6B2, 1:100, BioLegend) and Zombie Aqua (1:200, BioLegend) prepared in P2. Then, the cells were fixed with 2% paraformaldehyde and re-suspended in P2. Sample acquisition was done on an Aurora using SpectroFlo v2.2 (Cytek). Flow cytometry data were analyzed using FlowJo v10 (BD Biosciences). CD4 cells and CD8 were selected as live, singlet, and B220- cells.

### QUANTIFICATION AND STATISTICAL ANALYSES

Statistical significance was assigned when *P* values were < 0.05 using GraphPad Prism version 9.3. Tests, number of animals, median and geometric mean values, and statistical comparison groups are indicated in the Figure legends. Analysis of weight change was determined by two-way ANOVA. Changes in infectious virus titer, viral RNA levels, or serum antibody responses were compared between all conditions, and were analyzed by one-way ANOVA with multiple comparisons correction on ln-transformed data. Pairwise comparisons were done using a pairwise t-test.
